# Comparison of the Rhizosphere Bacterial Communities of Zigongdongdou Soybean and a High-Methionine Transgenic Line of This Cultivar

**DOI:** 10.1371/journal.pone.0103343

**Published:** 2014-07-31

**Authors:** Jingang Liang, Shi Sun, Jun Ji, Haiying Wu, Fang Meng, Mingrong Zhang, Xiaobo Zheng, Cunxiang Wu, Zhengguang Zhang

**Affiliations:** 1 Department of Plant Pathology, College of Plant Protection, Nanjing Agricultural University, and Key Laboratory of Integrated Management of Crop Diseases and Pests, Ministry of Education, Nanjing, China; 2 The National Key Facility for Crop Gene Resources and Genetic Improvement (NFCRI), MOA Key Laboratory of Soybean Biology (Beijing), Institute of Crop Science, The Chinese Academy of Agricultural Sciences, Beijing, China; 3 Nanchong Academy of Agricultural Science, Nanchong, China; Institute for Sustainable Plant Protection, C.N.R., Italy

## Abstract

Previous studies have shown that methionine from root exudates affects the rhizosphere bacterial population involved in soil nitrogen fixation. A transgenic line of Zigongdongdou soybean cultivar (ZD91) that expresses *Arabidopsis* cystathionine γ-synthase resulting in an increased methionine production was examined for its influence to the rhizosphere bacterial population. Using 16S rRNA gene-based pyrosequencing analysis of the V4 region and DNA extracted from bacterial consortia collected from the rhizosphere of soybean plants grown in an agricultural field at the pod-setting stage, we characterized the populational structure of the bacterial community involved. In total, 87,267 sequences (approximately 10,908 per sample) were analyzed. We found that Acidobacteria, Proteobacteria, Bacteroidetes, Actinobacteria, Chloroflexi, Planctomycetes, Gemmatimonadetes, Firmicutes, and Verrucomicrobia constitute the dominant taxonomic groups in either the ZD91 transgenic line or parental cultivar ZD, and that there was no statistically significant difference in the rhizosphere bacterial community structure between the two cultivars.

## Introduction

The global commercial cultivation of transgenic crops has increased from 1.7 million hectares in 1996 to 170.3 million hectares in 2012 [1]. Modern agricultural biotechnology and genetic engineering have allowed the development of crops with improved properties, such as the transgenic high-methionine soybean line Zigongdongdou 91 (ZD91) [2]. Most studies to date have suggested that the release of transgenic plants results in only minor changes to the microbial community structure and that these minor changes are often transient and short lived [3]–[15].

Soil microorganisms are affected by soil characteristics, environmental conditions, and crop management strategies [16]–[19]. Organic compounds in root exudates and rhizosphere microbial communities perform fundamental processes that contribute to nutrient cycling and plant health and root growth, which are governed by complex interactions driven by various factors such as soil types and plant species [3], [20]–[23]. The type and amount of root exudation may be an inherent property of a plant, and the rhizosphere microbial community is also expected to be unique [3], [24]–[26]. Thus, an altered exudate composition may result in a corresponding shift in the rhizosphere microorganism community [27]–[30]. According to Faragova et al. [31], genetically modified plants release root exudates different from those of non-transgenic counterparts, therefore affecting the microbial community of the rhizosphere. This occurs even if the genetically modified plant is of different cultivars of the same plant species [32]–[34]. It was reported that methionine has the effect of inhibiting nitrification in soil, and transgenic alfalfa that produces high levels of methionine has a profound effect on bacteria population involved in the nitrogen cycle of the soil [31], [35]. In a recent comparative study that employed Polymerase Chain Reaction-Denaturing Gradient Gel Electrophoresis (PCR-DGGE) technology to examine the bacterial communities in the rhizosphere of a transgenic high-methionine soybean, we found that the growth stage was the main factor affecting the community structure of the soil bacteria [36]. This finding is consistent with that of van Overbeek and van Elsas [25], Jin et al. [37] and Inceoglu et al. [38], in which bacterial community structures vary per growth stage changes. In addition, a study of rape (*Brassica napus*) indicated that the rhizosphere bacterial composition of a transgenic cultivar could be distinguished from that of its non-transgenic cultivar [39]. Conversely, in a study of potatoes, the microbial community structure in the rhizosphere was not significantly affected by genetic engineering [40], [41].

The biological nitrogen cycle is one of the most important nutrient cycles in the terrestrial ecosystem. It includes four major processes: nitrogen fixation, mineralization (decay), nitrification and denitrification. Studies have shown that there are three functional genes: *nifH*, *amoA*, and *nosZ*, which encode the key enzymes involved in nitrogen fixation, ammonia oxidization and complete denitrification, respectively. Microorganisms capable of nitrogen fixation are called diazotrophs whose composition is determined by associated plant species. In addition, agricultural practices can also influence soil diazotrophs [42]–[45]. There are two types of diazotrophs: free-living and symbiotic [46], [47]. The Rhizobia, *Frankia*, and Cyanobacteria are three important symbiotic diazotrophs. Rhizobia make up a paraphyletic group that falls into two classes in the phylum Proteobacteria: Alphaproteobacteria and Betaproteobacteria. Nitrification, which converts ammonium to nitrate, includes two steps: ammonia oxidation to nitrite, and nitrite oxidation to nitrate. The oxidation of ammonia to nitrite is performed by two groups of organisms, ammonia-oxidizing bacteria (AOB) and ammonia-oxidizing archaea (AOA) [48]. AOB is found among the Betaproteobacteria and Gammaproteobacteria [49], [50]. Denitrification is a microbially-facilitated process of nitrate reduction. So far, more than 60 genera of denitrifying microorganisms have been identified [51].

High-throughput TAG-encoded FLX amplicon pyrosequencing (using 16S rRNA genes) is a great technical advance for microbial ecology studies [52]. This technique can produce ∼400,000–1,000,000 reads in a single 4 h run, with an accuracy of approaching 99%, and the tag sequence contains adequate information for taxonomic assignment [53]. Several recent studies have used this technique with the 16S rRNA gene to determine the microbial diversity in various environmental samples [3], [52]–[60]. Given the wealth of information available due to the deep sequencing of environmental DNA, it is possible to determine whether these lineages are ecologically coherent [3]. It also has been proposed that some taxa are extremely coherent ecologically. Such taxa can be said to follow an r- or K-type life strategy [61]–[63].

In the present study, we used 16S rRNA gene-based pyrosequencing to compare the community dynamics of bacteria inhabiting the rhizosphere of field-grown transgenic cultivar ZD91 during the pod-setting stage to those of the corresponding non-transgenic ZD plants. Our study showed a wide variety of rhizosphere bacterial population associated with these cultivars but there was no significant difference in the bacterial population between transgenic cultivar ZD91 and parental cultivar ZD.

## Results

### Quantification of *nifH*, *amoA*, and *nosZ* genes, and carbon, nitrogen concentrations in the rhizospheres

To examine the effect of transgenic soybean on the abundance of diazotrophs, ammonia-oxidizing bacteria, and denitrifiers, we used quantitative real-time PCR (qPCR) assays to quantify *nifH*, *amoA*, and *nosZ* genes to inform nitrogen-cycling related bacteria collected from the rhizosphere soil. The abundance of *nifH*, *amoA*, and *nosZ* genes between ZD and ZD91 showed no significant differences (Table 1).

**Table 1:** Changes in abundance of *nifH*, *amoA*, and *nosZ* genes (Log_10_ copies/g soil), and carbon (C), nitrogen (N) contents in rhizosphere soil (*P*<0.01).

	ZD	ZD91
*nifH*	9.05±0.02 A	9.06±0.12 A
*amoA*	8.03±0.02 A	8.13±0.01 A
*nosZ*	7.70±0.08 A	7.90±0.02 A
Total C (%)	1.68±0.02 A	1.65±0.03 A
Total N (%)	0.06±0.01 A	0.07±0.01 A

We also estimated whether there are any differences in carbon and nitrogen levels in the rhizosphere of ZD and ZD91 and found no significant differences (Table 1).

### Amino acids in root exudates

Amino acids included aspartic acid, threonine, serine, glutamic acid, glycine, alanine, valine, methionine, isoleucine, leucine, tyrosine, phenylalanine, lysine and histidine in root exudates were determined. However, there were no differences in amino acids contents between ZD and ZD91 (Table 2).

**Table 2:** Amino acids secreted by soybean roots in the root box at pod-setting stage (µmol/ml) (*P*<0.01).

	ZD	ZD91
Asp	3.44±1.56 A	1.13±0.48 A
Thr	2.85±1.28 A	0.99±0.41 A
Ser	12.13±6.05 A	4.11±1.82 A
Glu	2.31±0.59 A	1.30±0.23 A
Gly	4.83±2.43 A	1.60±0.86 A
Ala	3.63±1.72 A	1.32±0.51 A
Val	2.14±0.83 A	1.01±0.25 A
Met	0.32±0.28 A	0.31±0.27 A
Ile	1.04±0.41 A	0.40±0.17 A
Leu	1.54±0.55 A	0.77±0.24 A
Tyr	2.03±0.58 A	1.33±0.20 A
Phe	1.22±0.47 A	0.65±0.17 A
Lys	1.03±0.57 A	0.33±0.20 A
His	2.12±1.36 A	0.68±0.46 A

### Pyrosequencing analysis of the bacterial communities of the rhizospheres

We performed 16S rRNA gene-based pyrosequencing analysis of the V4 region to characterize the bacterial community composition in the rhizospheres surrounding ZD and ZD91 cultivars. A total of 87,267 high-quality sequences was obtained, with an average of 10,908 sequences per sample. The results were summarized in Table 3 with detailed sample characteristics shown in Table S1. We used the grouping of cultivars based on the number of replicates (ZD, four replicates: ZD_1, ZD_2, ZD_3, and ZD_4; ZD91, four replicates: ZD91_1, ZD91_2, ZD91_3, and ZD91_4).

**Table 3:** Pyrosequencing data summary.

Sample	Sequences	OTUs	ACE	Chao	Shannon	Simpson	Coverage
ZD	10534±1353A	3391±505A	8900±1019A	6381±780A	7.34±0.21A	0.002±0.001A	0.82±0.02A
ZD91	11283±1818A	3649±370A	9637±795A	6911±688A	7.47±0.17A	0.001±0.000A	0.82±0.03A

Values are the mean±standard deviation of four replicates. Within each vertical column, values followed by the same letter are not statistically different from each other (*P*<0.01). The number of OTUs, richness estimators Chao and ACE, diversity estimators Shannon and Simpson, and Good’s coverage were calculated at 3% distance.

**Figure 1:**
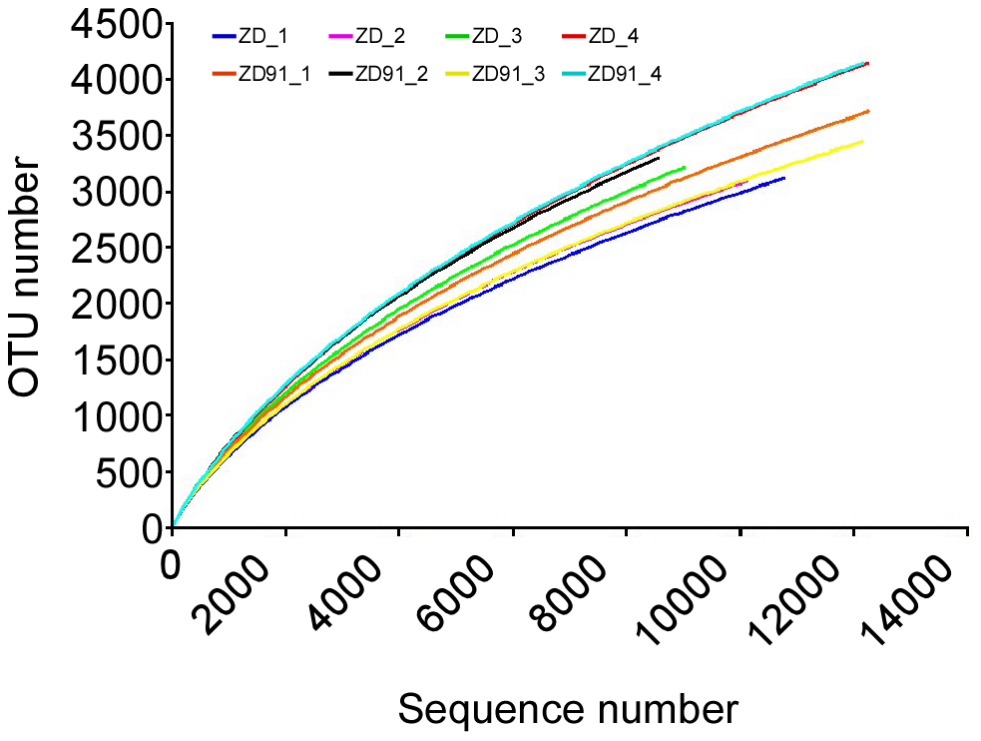
Rarefaction analysis. Rarefaction curves of OTUs clustered at 97% sequence identity across the samples. The sample labeled with ZD_1, ZD_2, ZD_3 and ZD_4 correspond to four replicates of cultivar ZD; ZD91_1, ZD91_2, ZD91_3 and ZD91_4 represent four replicates of transgenic cultivar ZD91.

The rarefaction curves tended towards the saturation plateau while Good’s coverage estimations revealed that 78–84% of the species were from eight samples, indicating that the sequence coverage was sufficient to capture the diversity of the bacterial communities in the samples (Fig. 1). The estimators of community richness (Chao and ACE) and diversity (Shannon and Simpson) were shown in Table 3, and there were no significant differences in these indices between ZD and ZD91. The null hypothesis was tested through AMOVA analysis (Table 4) and it did show that there were no significant differences between ZD and ZD91.

**Table 4:** AMOVA analysis between ZD and ZD91.

	ZD91-ZD	Among	Within	Total
Based on OTU (Jclass distance coefficient)	SS	0.261484	1.603340	1.86483
	df	1.000000	6.000000	7.00000
	MS	0.261484	0.267224	
	Fs: 0.978522			
	*p*-value: 0.745			
Based on OTU (Thetayc distance coefficient)	SS	0.0321963	0.290100	0.322296
	df	1.0000000	6.000000	7.000000
	MS	0.0321963	0.048350	
	Fs: 0.665901			
	*p*-value: 0.565			
Based on weighted UniFrac distances	SS	0.0172751	0.1477470	0.165022
	df	1.0000000	6.0000000	7.000000
	MS	0.0172751	0.0246245	
	Fs: 0.701539			
	*p*-value: 0.861			

Experiment-wise error rate: 0.05.

### Taxonomic composition

**Figure 2:**
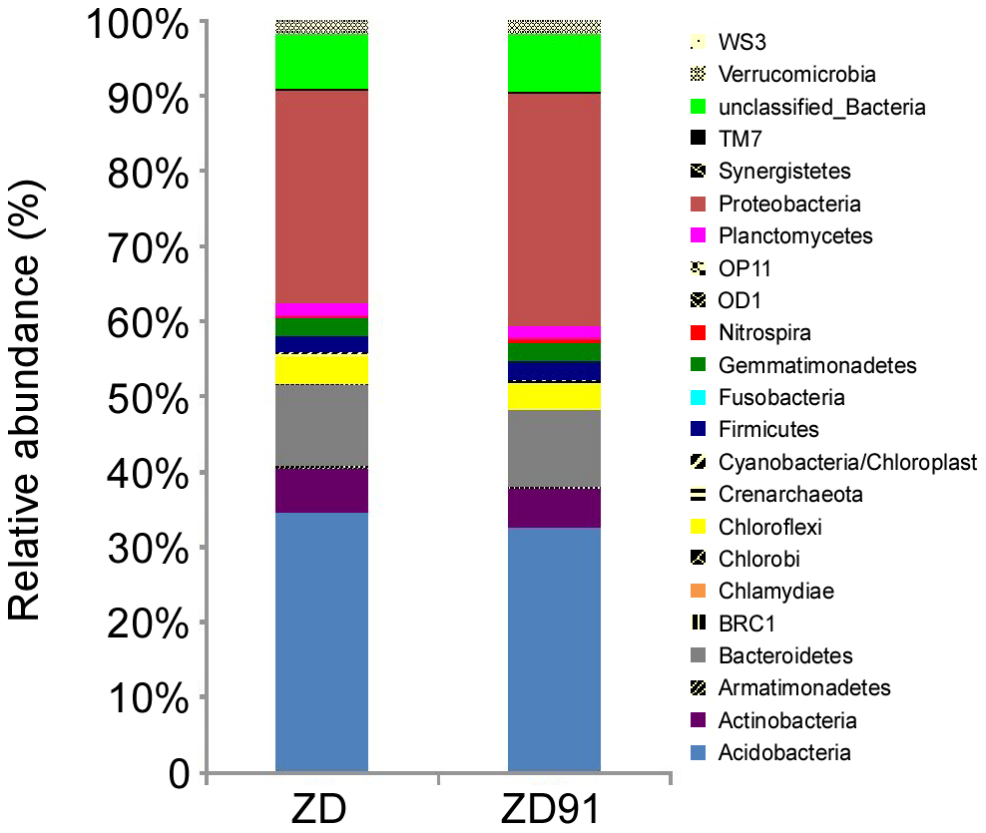
Bacterial composition at the phylum level. Relative read abundance of bacterial phyla within the communities. Sequences that could not be classified into any known group were assigned as unclassified_Bacteria.

**Figure 3:**
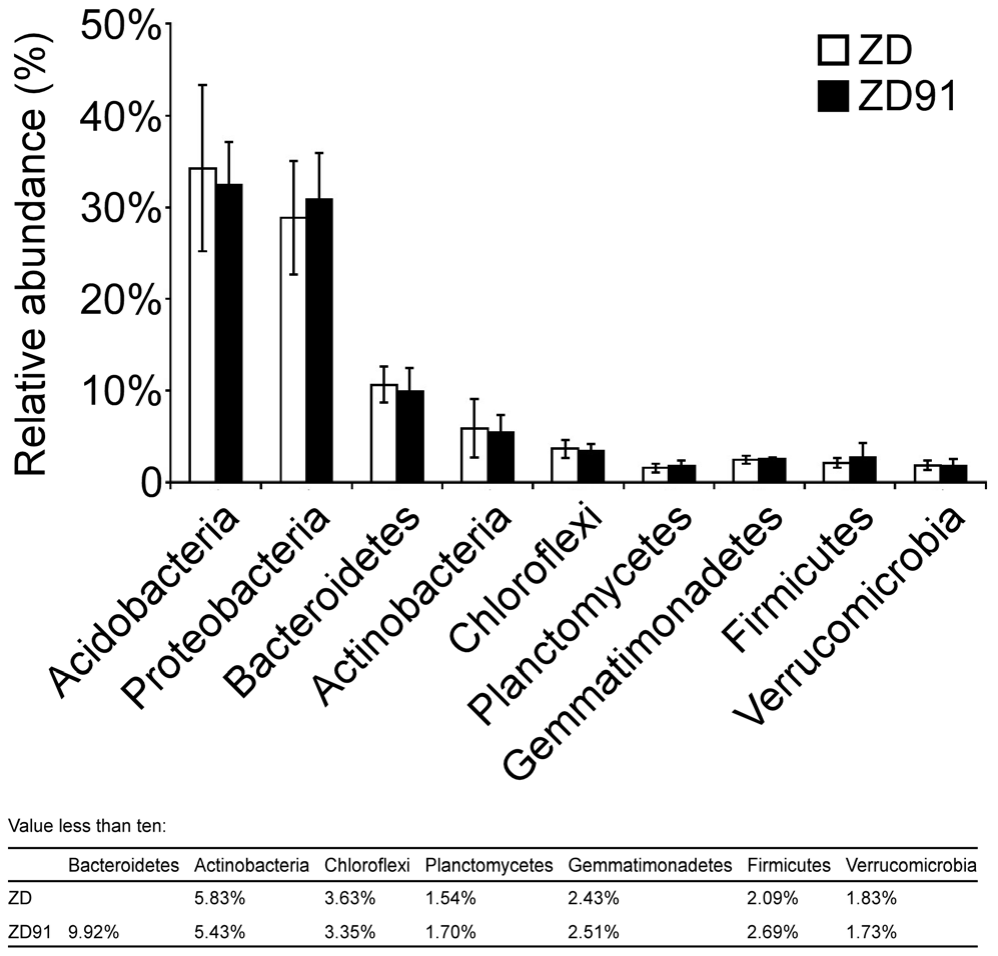
Relative abundance of main phyla in the rhizosphere of soybean cultivars. Error bars indicate standard errors. The *p*-values of the nine groups of bacteria, in order, were 0.734, 0.633, 0.662, 0.834, 0.687, 0.690, 0.751, 0.493, and 0.840 when testing for differences between cultivar ZD and transgenic cultivar ZD91.

Although probably present, this study did not attempt to characterize any Archaea. All sequences were classified from phylum to genus according to the RDP classifier using the default settings. 22 different phyla, including 1 archaeal phylum, were identified from the samples. The overall microbiota structure for ZD and ZD91 at the phylum level was shown in Figure 2 and Table S2. Acidobacteria, Proteobacteria, Bacteroidetes, Actinobacteria, Chloroflexi, Planctomycetes, Gemmatimonadetes, Firmicutes, and Verrucomicrobia accounted for >90% of the reads. Again, there were no observable differences among these categories between ZD and ZD91 at this taxonomic level (Fig. 3).

**Figure 4:**
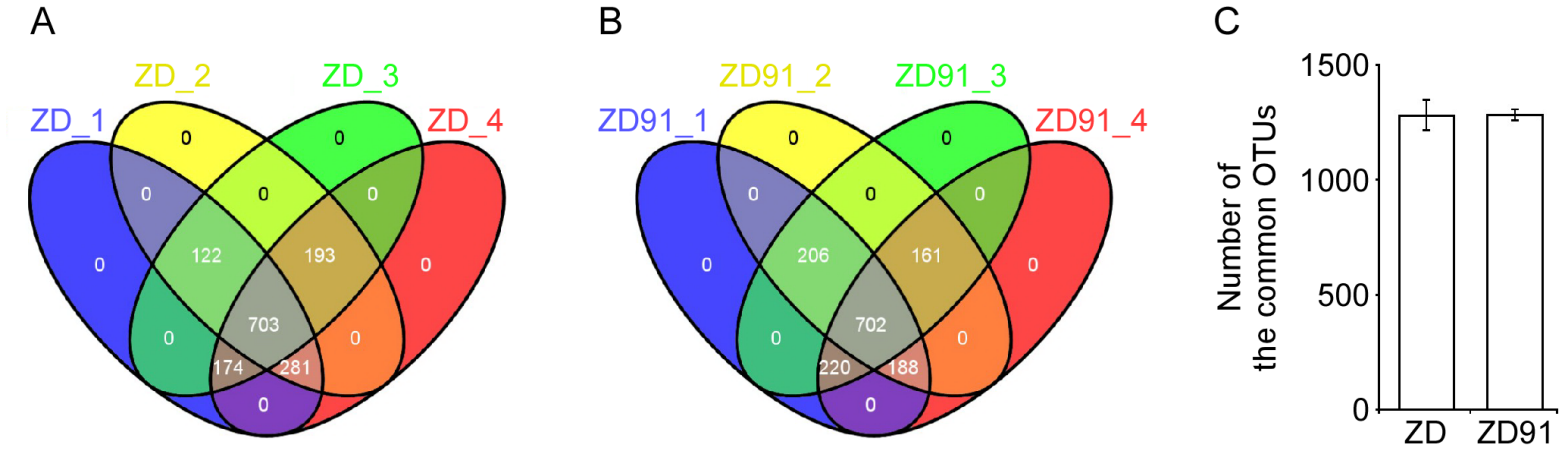
Common OTUs recovered from soil. (A) Venn diagram showing variable overlap between four replicates of ZD. (B) Venn diagram showing variable overlap between four replicates of ZD91. (C) Number of the common OTUs between ZD and ZD91.

A total of 13,299 operational taxonomic units was found in the complete data set (Table S3). The ten most abundant OTUs within each sample were identified (Table S4). There were 12 dominant OTUs in total, including Gp4, Gp6, *Terrimonas*, *Sphingosinicella, Sphingomonas, Flavisolibacter, Sphingomonadaceae, Lysobacter, Chryseobacterium, Acinetobacter, Levilinea and Opitutus.* A total of 2047 common OTUs was determined in rhizosphere soil and there was no significant difference between ZD and ZD91 (*P*<0.01) (Fig. 4). The ten most abundant genera from each of the eight samples were classified as a percentage of the total sequences per sample (Table S5). This analysis showed that there were 17 dominant genera in total, including four members of the Acidobacteria, five of the Bacteroidetes, three Alphaproteobacteria, two Gammaproteobacteria, and one each of the Actinobacteria, Gemmatimonadetes and Verrucomicrobia.

To analyze whether the distribution of low-abundance genera differs between samples, sequences with abundances below 0.1% were analyzed. A total of 481 such low-abundance genera was found. Of the 481 genera, 214 (70.63%), 203 (68.35%), 200 (61.54%), and 219 (64.60%) were observed in ZD_1, ZD_2, ZD_3, and ZD_4, respectively. In comparison, 208 (67.97%), 205 (61.75%), 219 (68.65%), and 206 (65.19%) were detected in ZD91_1, ZD91_2, ZD91_3, and ZD91_4, respectively. There were no genera unique to cultivar ZD or cultivar ZD91. Rare genera (abundances below 0.1%) accounted for over 60% in all samples, and there was no significant difference between cultivar ZD (66.28±4.02%) and cultivar ZD91 (65.89±3.14%).

Root-colonizing pseudomonads are known to be involved in plant growth and health. These bacteria increase plant growth either directly through the production of phytohormones or other stimulants and increasing the bioavailability of nutrients in the soil, or indirectly by the suppression of plant diseases and the induction of systemic resistance in plants [10]. A total of 6 pseudomonad species was found in our samples and there was no significant difference between ZD (0.07±0.03%) and ZD91 (0.05±0.04%).

### Nitrogen-fixing Bacteria

In the present study, *Frankia* was not found in any samples. Sequences related to Cyanobacteria, Alphaproteobacteria, and Betaproteobacteria were not significantly different between cultivar ZD (0.22±0.07%, 14.04±5.07%, and 1.23±0.30%) and cultivar ZD91 (0.14±0.08%, 13.56±4.88%, and 1.11±0.25%) samples.

### Nitrifying bacteria

In the present study, there were 35 Gammaproteobacteria genera detected. There was no significant difference between the sequences for cultivar ZD (3.47±0.17%) and ZD91 (4.49±1.38%). Nitrifying bacteria, which oxidize nitrite, include *Nitrobacter*, *Nitrospina*, *Nitrococcus*, and *Nitrospira* [65], but only *Nitrospira* was detected in this study. In addition, there was no significant difference between the sequences for cultivar ZD (0.36±0.16%) and cultivar ZD91 (0.44±0.16%).

### Denitrifying bacteria

In the present study, 36 such bacterial genera were found. The sequences related to denitrifying bacteria were not significantly different between cultivar ZD (3.13±1.31%) and cultivar ZD91 (2.80±0.28%).

### Shared OTUs in ZD and ZD91 libraries

The bacterial OTUs in the cultivar ZD and cultivar ZD91 libraries were further investigated for shared OTUs. As shown in Table S6, ZD_1, ZD_2, ZD_3, and ZD_4 had 700 OTUs in common. A statistical analysis revealed that the OTUs common to the four libraries comprised 58.96%, 59.62%, 52.19%, and 46.82% of the reads in the ZD_1, ZD_2, ZD_3, and ZD_4 libraries, respectively. Acidobacteria, Proteobacteria, and Bacteroidetes comprised 531 of the shared OTUs (75.86% in proportion). Further, the OTUs common to the four libraries of the transgenic cultivar comprised 54.04%, 49.01%, 52.17%, and 42.50% of the reads in the ZD91_1, ZD91_2, ZD91_3, and ZD91_4 libraries, respectively. Acidobacteria, Proteobacteria, and Bacteroidetes included 534 of the shared OTUs (76.29% in proportion).

### Bacterial community dynamics

**Figure 5:**
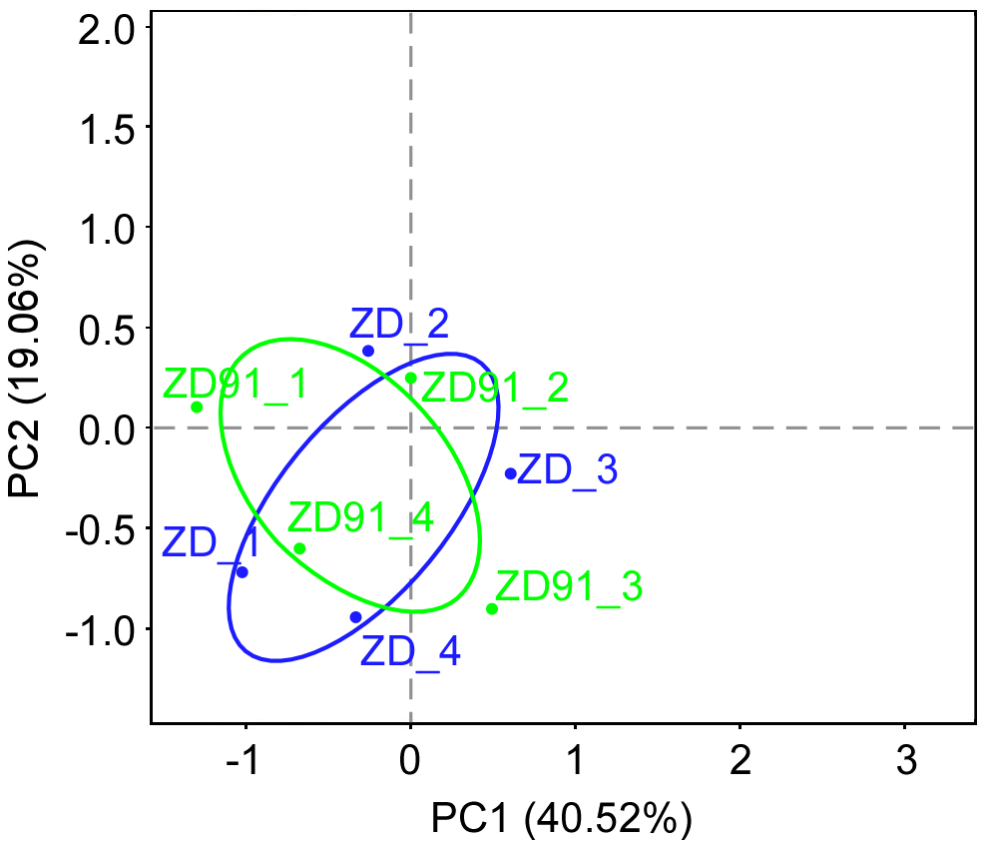
Principal coordinate analysis (PCoA) plot of samples using the weighted UniFrac distance metric. The variance explained by each principal coordinate axis is shown in parentheses. Datasets were subsampled to equal depth prior to the UniFrac distance computation. A circle was drawn with R around the samples of the same type (blue represents cultivar ZD, and green represents transgenic cultivar ZD91).

To examine the effect of cultivar on the total distribution of phyla and genera, we performed principal coordinate analysis (PCoA) of our data. The PCoA analysis of weighted UniFrac distances was used to calculate pairwise distances between the rhizosphere bacterial communities of the eight samples [64]. The first two axes of the PCoA represented 40.52% and 19.06% of the total variation, respectively (Fig. 5). AMOVA analysis (Table S7) was used to find out whether the separation of cultivar ZD and cultivar ZD91 in the PCoA was statistically significant. The result showed *p*-value was 0.859 indicating no significantly different between cultivar ZD and cultivar ZD91. Furthermore, we calculated ADONIS differences between cultivar ZD and cultivar ZD91 (Table 5), and found no significant difference between these two cultivars. Thus, the bacterial communities in the cultivars were not different from each other.

**Table 5:** ADONIS differences between ZD and ZD91.

	Df	SumsOfSqs	MeanSqs	F. Model	R^2^	Pr(>F)
qiime.data$map [[opts$category]]	1	0.017275	0.017275	0.70124	0.10464	0.8492
Residuals	6	0.147814	0.024636		0.89536	
Total	7	0.165089			1.00000	

ADONIS differences is a non-parametric method to measure statistical significance of sample grouping. It was calculated based on weighted UniFrac distance metric.

## Discussion

In this study, we monitored the quantitative changes of the key genes involved in N-cycling as well as elements carbon, nitrogen concentrations in rhizosphere soil of ZD and ZD91, and analyzed the amino acids contents in the root exudates of different cultivars. We found that there were no significant differences between cultivar ZD and cultivar ZD91 in nitrogen-cycling genes and carbon, nitrogen concentrations. The results showed that the amino acids contents in the root exudates of ZD91 were lower than ZD, but they were not statistically significant.

We assessed the dynamics of the relative soil abundance of bacteria and the structure of soil bacterial communities as a function of cultivar type using the transgenic soybean cultivar ZD91 and its parental non-transgenic soybean cultivar ZD in a single experimental field. According to the pyrosequencing results, genetic modification via the insertion and expression of *AtD*-*CGS* did not affect the composition of the bacterial community in the rhizosphere of soybean. However, the profiles of independent replicates differed in terms of the abundance of various phyla (Fig. 5 and Table S2), but this variation did not correlate with the experimental variables. All plots were on the same field, but due to the large size of the field, the replicates were located an average of 20 meters apart from each other. Thus, field heterogeneity was not considered when evaluating the impact of transgenic soybean on rhizosphere bacterial community. Furthermore, we analyzed the OTUs of high abundances and repeated well (occurred in more than three samples, defined as the common OTUs) in the soil. Since the singletons and low abundance OTUs were detected randomly, and there was no significant difference within the treatment (among the 4 replicates) and between the treatments (Fig. 4).

Due to logistic and technical (e.g., low sequence numbers) constraints, studies of soil microbial diversity and function have focused mainly on the most dominant species [3]. Previous studies have demonstrated weaknesses in traditional molecular methods (e.g., DGGE, RFLP, ARDRA, and SSCP) in terms of resolution; specifically, it is difficult to detect subtle changes in banding patterns or changes in closely related species, leading to the underestimation of bacterial diversity by one to two orders of magnitude [4], [54], [57], [66]. Moreover, some rare species may have large effects on soil function, in spite of their low total biomass or rarity [67], [68]. In this study, pyrosequencing provided a promising, fast, economical, and unique opportunity to access the less abundant bacterial taxa surrounding soybean plants, as well as the chance to compare their presence across cultivars in the pod-setting stage [54], [55], [57]. Similar to the results of Liu et al. [20] and Xu et al. [21], [69], our sequencing results revealed that numerous bacterial phyla (e.g., Acidobacteria, Proteobacteria, Bacteroidetes, Actinobacteria, Chloroflexi, Planctomycetes, Gemmatimonadetes, Firmicutes, Nitrospirae, Verrucomicrobia, BRC1, Chlamydiae, Cyanobacteria, Fusobacteria, Chlorobi, OD1, OP11, Synergistetes, TM7, Armatimonadetes, and WS3) commonly inhabit the soybean rhizosphere.

Moreover, dominant members of particular phyla or classes were used as indicators to reveal the prevailing ecological pattern (e.g., along the copiotroph/oligotroph scale) [3]. Members of the Betaproteobacteria and Bacteroidetes, as well as species belonging to the genus *Pseudomonas*, tend to be favored in r-selection/copiotrophic soils, which have higher carbon availability, while Acidobacteria are favored under K-selection/oligotrophic conditions [61]–[63]. Consequently, such groups may have higher abundances in soils with higher versus lower levels of easily available carbon, respectively. Furthermore, the ratio of Proteobacteria to Acidobacteria can be used as an indicator of the soil trophic level [70]. Although it is rather implausible that a whole phylum would have shared ecological characteristics [3], our indicators revealed variability (based on the ratio of Proteobacteria to Acidobacteria) occurs in the response of Proteobacteria versus Acidobacteria to shifting ecological conditions in the soil. The ratio is 0.16 in oligotrophic soil [71], [72], 0.34 in low-input agricultural soil [73], 0.46 in low-nutrient systems [62], and 0.87 in high-input agricultural systems [74]. The values obtained in this study, 0.93±0.46 in cultivar ZD, and 0.98±0.31 in cultivar ZD91, indicating a high-nutrient system, but again there was no significant difference between the two cultivars.

The pyrosequencing protocol used in previous investigations and here carries certain limitations [3], [52]–[60]. Any PCR primer set for the amplification of 16S rDNA genes may miss a considerable amount of the extant microbial diversity; thus, at present, no clear answer can be given with respect to the real extant community make-up [3]. Because PCR biases mostly affect the detection of rare sequences in a sample, it is important to view such data on the rare biosphere with proper cautions.

Of the ten most abundant bacterial OTUs in the samples, several were related to Acidobacteria. Of the ten most abundant bacterial genera, several were related to both Acidobacteria and Proteobacteria. The Acidobacteria are physiologically diverse and ubiquitous, especially in soil, but are underrepresented in culture [75]–[77]. Because most Acidobacteria have not been cultured, their ecology and metabolism are not well understood [76]. However, these bacteria may be important contributors to ecosystems because they are particularly abundant in soil [78]. The Proteobacteria are a major group of bacteria, and include many of the species responsible for nitrogen fixation.

We also monitored structural and quantitative changes in the bacterial communities of each soybean regarding nitrogen cycling. The abundance of nitrogen-transforming bacteria were not significantly different between cultivars ZD and ZD91.

In conclusion, we found that 454-pyrosequencing can be a useful tool for risk assessment studies to analyze the immediate impact of plants on the diversity of soil bacteria in rhizospheres. Of all DNA reads, 7.56% belonged to unclassified bacteria, containing a suite of diverse taxa. To understand the ecological impact of unclassified sequences, they must be identified and ideally an organism should be cultured. This once again stresses that culture-independent techniques should go hand-in-hand with culture-dependent ones [3]. For ecological risk identification, however, the observation of shifts during one stage of plant development and in a one-year period is insufficient. The positive identification of risk requires that changes be demonstrated at all stages of soybean development and over an extended time frame. The fact that no effect between transgenic and non-transgenic plants was detected suggests only minor physiological changes caused by the insertion of *AtD*-*CGS* into the soybean genome.

## Materials and Methods

### Soybean Cultivars

Transgenic soybean cultivar (ZD91) contains the *Arabidopsis* cystathionine γ-synthase (*AtD*-*CGS*) gene which has been introduced artificially into the soybean cultivar Zigongdongdou (ZD) using *Agrobacterium*-mediated transformation, and which exhibits a high content of methionine in the seeds.

### Field tests setup and sampling

An experimental field - Nanchong (30°48′N, 106°04′E), Sichuan Province, China, in which a completely randomized block design was set out in 2011, was used. The basic properties of the soil were 46.98 g/kg organic matter, 0.51 g/kg total nitrogen, 11.73 mg/kg available phosphorus, 220.40 mg/kg available potassium, and 52.81 mg/kg alkali-hydrolyzed nitrogen. The studied region was 2.8 acres. For each soybean cultivar, four replicate plots, which were randomly distributed over the field, were used. The field was under standard agricultural practice. The samples were collected at the pod-setting stage (80 days after seedling emergence) of the two soybean cultivars. Rhizosphere soil samples were collected as described previously [38]. Briefly, five plants were carefully sampled using five-point sampling method and the plants with adhering soil were immediately taken to the laboratory. The loosely adhering soil on the roots was shaken off, and the resulting roots (containing rhizosphere soil) were pooled per plot. Using the pooled sample, soil tightly adhering to the roots was brushed off and collected (constituting rhizosphere soil).

### Ethics statements

This study was approved by the Ministry of Agriculture of the People’s Republic of China and the genetically modified organisms safety team of Nanjing Agricultural University, China. The field studies did not involve endangered or protected species. The land was not privately owned or protected in any way.

### Soil DNA extraction, PCR amplification and pyrosequencing

Soil DNA was extracted from rhizosphere soil samples by employing the FastDNA SPIN Kit for Soil (MP Biomedicals, USA) as recommended by the manufacturer. DNA concentrations were quantified by using a NanoDrop 1000 Spectrophotometer (Thermo Scientific, USA) according to the manufacturer’s protocol. The 16S rDNA gene (V4 region) was amplified using the barcode primers 515F [79] and 926R [80]. The primers were designed as 5′-adapter+barcode+gene specific primer-3′ (Table S8). The PCR reaction mixture (20 µL) contained 2 µL 10-fold buffer, 1.6 µL dNTP mix (2.5 mM each), 1 µL BSA (20 mg/ml), 0.25 µL rTaq polymerase (2.5 U/µL), 0.2 µL of each of the primers (10 µM), 0.5–5 µL (1–50 ng) of isolated DNA as template. The mixtures were placed in a TaKaRa PCR Thermal Cycler Dice (TaKaRa, Japan) and thermal cycling was performed as follows: initial denaturation consisting of 5 min at 95°C; followed by 30 cycles consisting of 30 sec at 94°C, 30 sec at 57°C and 30 sec at 72°C; and final extension for 10 min at 72°C. Analysis of PCR products on 1% agarose gel revealed bands of the corresponding size. These bands were cut and purified using the QIAquick Gel Extraction Kit as recommended by the manufacturer (Qiagen, Netherlands), and then purified by Agencourt AMPure XP (Beckman Coulter, USA). Quantification of the purified PCR products was performed using the Quant-iT PicoGreen dsDNA Assay Kit (Invitrogen, Netherlands) and a TBS-380 Fluorometer (Turner Biosystems, USA) as recommended by the manufacturer. The PCR products from different samples were then mixed in equal ratios (7.2 ng per sample) for pyrosequencing with a Roche Genome Sequencer GS-FLX Titanium platform [56].

### Real-time PCR

The abundance of *nifH*, *amoA*, and *nosZ* genes in all samples were quantified using real-time PCR. Quantitative real-time PCR was performed according to the methods from previous studies: *nifH* (as a measure of N-fixing bacteria) used primers nifH-F (5′-AAA GGY GGW ATC GGY AAR TCC ACC AC-3′) and nifH-R (5′-TTG TTS GCS GCR TAC ATS GCC ATC AT-3′) [81]; *amoA* (as a measure of ammonia-oxidizing bacteria) used primers amoA-1F (5′-GGG GTT TCT ACT GGT GGT-3′) and amoA-2R (5′-CCC CTC KGS AAA GCC TTC TTC-3′) [82]; and *nosZ* (as a measure of denitrification bacteria) used primers nosZ-F (5′-CGY TGT TCM TCG ACA GCC AG-3′) and nosZ 1622R (5′-CGS ACC TTS TTG CCS TYG CG-3′) [83]. Purified PCR products from a common DNA mixture (equal amounts of DNA from all samples) were used to prepare sample-derived quantification standards as previously described [84]. In comparison to using a clone (plasmid) as standard, this method avoids the difference of PCR amplification efficiency between standards and samples caused by the different sequence composition in the PCR templates (single sequence in a plasmid for the standard vs. mixture of thousands of sequences in a soil sample) [83]. The copy number of gene in each sample was calculated by http://cels.uri.edu/gsc/cndna.html and the data were Log_10_ transformed [84]. The 20 µL reaction mixture contained 10 µL SYBR *Premix Ex Taq* (Tli RNaseH Plus) (2×) (TaKaRa, Japan), 0.4 µL of each primer (10 µM), 0.4 µL ROX Reference Dye II (50×) (TaKaRa, Japan), 2 µL template, and 6.8 µL dH_2_O (sterile distilled water). Real-time amplification was performed in an Applied Biosystems 7500 Fast Real-Time PCR System using the following program: 95°C for 30 s; 40 cycles of 95°C for 5 s, (58°C for *nifH* gene; 57°C for *amoA* gene; 64°C for *nosZ* gene) for 31 s, 72°C for 34 s. A dissociation step was added at the end of the qPCR to assess amplification quality. The R^2^ of all these standard curves were higher than 0.99. All quantitative PCR reactions were performed in triplicate.

### Root box design and determination of amino acids secreted by roots

The root box used in this study was designed as reported previously [85]. There were three replicates of root box for each genotype. The lower chamber of the root box was filled with 500 mL of sterile ultrapure water, which was used to collect the amino acids exuded by the roots during a 72-h period at the pod-setting stage. Samples of root exudate were collected, filtered through a 0.45 µm membrane, freeze-dried, and redissolved in 1 mL of deionized water. Amino acids contents in the root exudates were analyzed by L-8900 Amino Acid Analyzer (Hitachi, Japan).

### Chemical analysis of carbon and nitrogen contents in rhizosphere soil

The concentrations of carbon and nitrogen in rhizosphere soil were determined by Vario MICRO cube (Elementar, Germany). The detection was based on JY/T 017–1996 General rules for elemental analyzer which was approved by Ministry of Education of the People’s Republic of China.

### Bioinformatic analysis

All sequences generated in this study can be downloaded from NCBI Sequence Read Archive, accession number: SRP026381. On the basis of several previous reports describing sources of errors in 454 sequencing runs, the valid reads should comply with the following rules: the sequences with low quality (length<200 bp, with ambiguous base ‘N’, and average base quality score<20) were removed; the sequences that matched the primer and one of the used barcode sequences and had at least an 80% match to a previously determined 16S rRNA gene sequence were retained [54], [56], [83], [86]. The sequences were used for chimera check using UCHIME method in Mothur [87], and chimeric sequences were removed.

The high-quality sequences were assigned to samples according to barcodes. Sequences were aligned in accordance with RDP (Ribosomal Database Project) alignment [88] and clustered into operational taxonomic units (OTUs). OTUs were defined using a threshold of 97% identity, within and between groups, which was a criterion for species level delineation in previous studies [56]. OTUs that reached 97% similarity level were used for diversity (Shannon and Simpson), richness (Chao and ACE), Good’s coverage, and Rarefaction curve analysis by using Mothur [87]. The OTUs which occurred in more than three samples from either ZD or ZD91 were defined as the common OTUs [89]. Taxonomic assignments of OTUs exhibiting 97% similarity were performed by using Mothur in accordance with RDP Classifier at 80% confidence threshold. The null hypothesis was tested using OTU and weighted UniFrac distances. The study objective was to test whether the transgenic soybean had an effect on rhizosphere bacterial communities. AMOVA analysis was done by using Mothur. ADONIS differences were calculated by using R.

To compare bacterial community structures across all samples based on the relative abundance of bacterial phyla, Principal coordinate analysis (PCoA) was performed by using Qiime [90]. AMOVA analysis was used to find out whether the separation of ZD and ZD91 in the PCoA was statistically significant.

### Statistical analysis

One-way analysis of variance and Duncan pair-wise comparisons (*P*<0.01) were used to determine the minimum significant difference between soybean cultivars (rhizosphere soils) by employing SPSS version 17.0 for Windows.

## Supporting Information

Table S1 **Pyrosequencing data and estimator index of each sample.** The number of OTUs, richness estimator Chao and ACE, diversity estimator Shannon and Simpson, and Good’s coverage were calculated at 3% distance.
(DOC)

Table S2 **Bacterial composition of the communities at the phylum level.** The number in the table (except the first line and column) represent pyrosequencing reads.
(DOC)

Table S3 **Different OTUs for each sample.**
(XLS)

Table S4 **Classification of the 10 most abundant bacterial OTUs in the eight samples.** Relative abundance (%, a percentage of the total sequences per sample) of each OTU is under the genus name/group number. OTUs were identified using 97% cutoffs. All Gp4 OTU classify in the same class ‘Acidobacteria_Gp4’ and all Gp6 OTU classify in the same class ‘Acidobacteria_Gp6’.
(DOC)

Table S5 **The ten most abundant genera found in the eight samples.** Relative abundance (%, a percentage of the total sequences per sample) of each genus is included in parentheses. All Gp6 classify in the same class ‘Acidobacteria_Gp6’, all Gp4 classify in the same class ‘Acidobacteria_Gp4’ and all Gp3 classify in the same class ‘Acidobacteria_Gp3’.
(DOC)

Table S6 **Shared OTUs in the samples.** (A) Shared OTUs in the ZD libraries. (B) Shared OTUs in the ZD91 libraries.
(DOC)

Table S7 **AMOVA analysis between ZD and ZD91 in PCoA.** AMOVA analysis in PCoA (classfily method: UPGMA). AMOVA analysis was used to find out whether the separation of ZD and ZD91 in the PCoA is statistically significant.
(DOC)

Table S8 **Primers with tags and adapters used in pyrosequencing.**
(DOC)
