# Supplementary material for: Comparison of the Rhizosphere Bacterial Communities of Zigongdongdou Soybean and a High-Methionine Transgenic Line of This Cultivar
Source: PLoS One. 2014 Jul 31;9(7):e103343. doi: 10.1371/journal.pone.0103343 (PMC4117502; doi:10.1371/journal.pone.0103343)
Supplement: Table S1 — Pyrosequencing data and estimator index of each sample. The number of OTUs, richness estimator Chao and ACE, diversity estimator Shannon and Simpson, and Good’s coverage were calculated at 3% distance. (DOC) [file pone.0103343.s001.doc]

**Table S1. Pyrosequencing data and estimator index of each sample**

| **Sample** | **Sequences** | **OTUs** | **ACE** | **Chao** | **Shannon** | **Simpson** | **Coverage** |
| --- | --- | --- | --- | --- | --- | --- | --- |
| ZD_1 | 10762 | 3115 | 8379.142 | 6021.211 | 7.131 | 0.003 | 0.839 |
| ZD_2 | 10115 | 3092 | 8197.623 | 5778.164 | 7.210 | 0.002 | 0.830 |
| ZD_3 | 9010 | 3212 | 8615.551 | 6203.223 | 7.394 | 0.001 | 0.796 |
| ZD_4 | 12250 | 4145 | 10406.692 | 7521.360 | 7.611 | 0.001 | 0.813 |
| ZD91_1 | 12248 | 3709 | 9770.430 | 7034.160 | 7.398 | 0.002 | 0.833 |
| ZD91_2 | 8556 | 3299 | 9161.126 | 6259.450 | 7.554 | 0.001 | 0.780 |
| ZD91_3 | 12174 | 3445 | 8914.841 | 6526.820 | 7.272 | 0.002 | 0.845 |
| ZD91_4 | 12152 | 4142 | 10699.766 | 7824.075 | 7.659 | 0.001 | 0.810 |

The number of OTUs, richness estimator Chao and ACE, diversity estimator Shannon and Simpson, and Good’s coverage were calculated at 3% distance.
