# Supplementary material for: Comparison of the Rhizosphere Bacterial Communities of Zigongdongdou Soybean and a High-Methionine Transgenic Line of This Cultivar
Source: PLoS One. 2014 Jul 31;9(7):e103343. doi: 10.1371/journal.pone.0103343 (PMC4117502; doi:10.1371/journal.pone.0103343)
Supplement: Table S2 — Bacterial composition of the communities at the phylum level. The number in the table (except the first line and column) represent pyrosequencing reads. (DOC) [file pone.0103343.s002.doc]

**Table S2. Bacterial composition of the communities at the phylum level**

| **phylum** | **ZD_1** | **ZD_2** | **ZD_3** | **ZD_4** | **ZD91_1** | **ZD91_2** | **ZD91_3** | **ZD91_4** |
| --- | --- | --- | --- | --- | --- | --- | --- | --- |
| Acidobacteria | 4763 | 3886 | 2107 | 3773 | 4664 | 2695 | 3241 | 4054 |
|  |  |  |  |  |  |  |  |  |
| Actinobacteria | 342 | 319 | 852 | 924 | 452 | 695 | 550 | 653 |
|  |  |  |  |  |  |  |  |  |
| Armatimonadetes | 31 | 24 | 44 | 55 | 35 | 35 | 24 | 28 |
|  |  |  |  |  |  |  |  |  |
| Bacteroidetes | 1232 | 929 | 808 | 1594 | 1318 | 581 | 1568 | 1122 |
|  |  |  |  |  |  |  |  |  |
| BRC1 | 9 | 2 | 4 | 1 | 2 | 2 | 5 | 2 |
|  |  |  |  |  |  |  |  |  |
| Chlamydiae | 1 | 1 |  | 1 | 5 | 6 | 12 | 3 |
|  |  |  |  |  |  |  |  |  |
| Chlorobi | 1 | 6 | 8 | 5 | 3 | 2 | 11 | 6 |
|  |  |  |  |  |  |  |  |  |
| Chloroflexi | 478 | 273 | 251 | 560 | 429 | 275 | 287 | 525 |
|  |  |  |  |  |  |  |  |  |
| Crenarchaeota | 24 | 16 | 28 | 57 | 12 | 34 | 28 | 43 |
|  |  |  |  |  |  |  |  |  |
| Cyanobacteria | 17 | 20 | 29 | 35 | 16 | 27 | 28 | 12 |
|  |  |  |  |  |  |  |  |  |
| Firmicutes | 164 | 181 | 245 | 283 | 224 | 428 | 211 | 265 |
|  |  |  |  |  |  |  |  |  |
| Fusobacteria | 2 |  |  |  | 2 | 2 |  | 5 |
|  |  |  |  |  |  |  |  |  |
| Gemmatimonadetes | 282 | 300 | 201 | 230 | 315 | 225 | 312 | 275 |
|  |  |  |  |  |  |  |  |  |
| Nitrospira | 21 | 53 | 43 | 32 | 37 | 57 | 44 | 50 |
|  |  |  |  |  |  |  |  |  |
| OD1 |  |  | 1 |  |  |  |  |  |
|  |  |  |  |  |  |  |  |  |
| OP11 | 1 | 1 |  | 1 |  |  |  | 3 |
|  |  |  |  |  |  |  |  |  |
| Planctomycetes | 93 | 168 | 144 | 249 | 111 | 196 | 194 | 245 |
|  |  |  |  |  |  |  |  |  |
| Proteobacteria | 2459 | 3024 | 3330 | 3131 | 3480 | 2469 | 4665 | 3363 |
|  |  |  |  |  |  |  |  |  |
| Synergistetes |  |  | 1 |  |  |  |  |  |
|  |  |  |  |  |  |  |  |  |
| TM7 | 12 | 40 | 36 | 22 | 27 | 14 | 33 | 15 |
|  |  |  |  |  |  |  |  |  |
| unclassified_Bacteria | 612 | 727 | 728 | 991 | 898 | 692 | 821 | 1126 |
|  |  |  |  |  |  |  |  |  |
| Verrucomicrobia | 207 | 132 | 146 | 301 | 202 | 112 | 133 | 348 |
|  |  |  |  |  |  |  |  |  |
| WS3 | 11 | 13 | 4 | 5 | 16 | 9 | 7 | 9 |

The number in the table (except the [first](app:ds:first) [line](app:ds:line) and column) represent pyrosequencing reads.
