# Supplementary material for: Comparison of the Rhizosphere Bacterial Communities of Zigongdongdou Soybean and a High-Methionine Transgenic Line of This Cultivar
Source: PLoS One. 2014 Jul 31;9(7):e103343. doi: 10.1371/journal.pone.0103343 (PMC4117502; doi:10.1371/journal.pone.0103343)
Supplement: Table S4 — Classification of the 10 most abundant bacterial OTUs in the eight samples. Relative abundance (%, a percentage of the total sequences per sample) of each OTU is under the genus name/group number. OTUs were identified using 97% cutoffs. All Gp4 OTU classify in the same class ‘Acidobacteria_Gp4’ and all Gp6 OTU classify in the same class ‘Acidobacteria_Gp6’. (DOC) [file pone.0103343.s004.doc]

Table S4. Classification of the 10 most abundant bacterial OTUs in the eight samples

| ZD_1 | ZD_2 | ZD_3 | ZD_4 | ZD91_1 | ZD91_2 | ZD91_3 | ZD91_4 |
| --- | --- | --- | --- | --- | --- | --- | --- |
| Gp4  (OTU24445)  3.17 | Gp4  (OTU24445)  2.21 | *Sphingomonas* (OTU22283)  1.49 | Gp4  (OTU24445)  1.24 | Gp4  (OTU24445)  2.16 | Gp4  (OTU24445)  0.94 | Gp4  (OTU24445)  1.15 | Gp4  (OTU24445)  1.02 |
|  |  |  |  |  |  |  |  |
| Gp4  (OTU24293)  1.31 | Gp4  (OTU24270)  0.95 | *Sphingosinicella* (OTU23552)  1.35 | Gp4  (OTU24735)  1.13 | Gp4  (OTU24270)  0.91 | Gp4  (OTU10917)  0.58 | *Chryseobacterium* (OTU24622)  1.11 | Gp6  (OTU10698)  0.81 |
|  |  |  |  |  |  |  |  |
| Gp4  (OTU24270)  0.97 | Gp4  (OTU24293)  0.86 | Gp4  (OTU24735)  0.80 | *Terrimonas* (OTU24578)  0.73 | *Sphingosinicella* (OTU23552)  0.79 | *Lysobacter* (OTU24767)  0.51 | *Sphingosinicella* (OTU23552)  0.97 | *Levilinea* (OTU24077)  0.53 |
|  |  |  |  |  |  |  |  |
| Gp4  (OTU10917)  0.96 | *Sphingosinicella* (OTU23552)  0.86 | Gp4  (OTU21500)  0.62 | Gp4  (OTU22386)  0.70 | Gp4  (OTU24293)  0.70 | Gp6  (OTU10698)  0.50 | *Sphingomonas* (OTU22283)  0.88 | Gp4  (OTU24293)  0.50 |
|  |  |  |  |  |  |  |  |
| Gp4  (OTU22933)  0.86 | *Sphingomonas* (OTU22283)  0.81 | Gp4  (OTU22386)  0.61 | Gp6  (OTU10698)  0.63 | *Sphingomonadaceae* (OTU24238)  0.63 | *Sphingosinicella* (OTU23552)  0.47 | *Acinetobacter* (OTU13482)  0.84 | *Opitutus* (OTU20149)  0.45 |
|  |  |  |  |  |  |  |  |
| Gp4  (OTU23507)  0.75 | Gp4  (OTU10917)  0.78 | Gp4  (OTU24445)  0.57 | Gp4  (OTU24211)  0.60 | Gp4  (OTU10917)  0.59 | Gp6  (OTU21829)  0.47 | *Flavisolibacter* (OTU22634)  0.81 | Gp4  (OTU24735)  0.42 |
|  |  |  |  |  |  |  |  |
| Gp4  (OTU24071)  0.71 | Gp4  (OTU22386)  0.70 | Gp4  (OTU22933)  0.55 | Gp4  (OTU24293)  0.50 | Gp4  (OTU23061)  0.58 | *Sphingomonas* (OTU22283)  0.46 | Gp4  (OTU24293)  0.67 | *Terrimonas* (OTU24578)  0.41 |
| Gp4  (OTU21034)  0.69 | Gp4  (OTU21497)  0.68 | *Sphingomonas* (OTU23760)  0.46 | Gp4  (OTU24270)  0.46 | *Sphingomonas* (OTU22283)  0.56 | *Flavisolibacter* (OTU22634)  0.44 | *Sphingomonas* (OTU24740)  0.67 | Gp4  (OTU10917)  0.40 |
|  |  |  |  |  |  |  |  |
| Gp4  (OTU21500)  0.68 | *Flavisolibacter* (OTU22634)  0.63 | Gp4  (OTU21497)  0.44 | Gp4  (OTU21034)  0.46 | *Sphingomonas* (OTU24191)  0.49 | Gp4  (OTU22933)  0.42 | *Sphingomonadaceae* (OTU24238)  0.62 | *Flavisolibacter* (OTU22634)  0.40 |
|  |  |  |  |  |  |  |  |
| *Terrimonas* (OTU22343)  0.57 | Gp4  (OTU22933)  0.54 | *Sphingomonas* (OTU24770)  0.44 | Gp4  (OTU10917)  0.45 | Gp4  (OTU22386)  0.49 | Gp4  (OTU24293)  0.41 | *Chryseobacterium* (OTU23392)  0.61 | Gp4  (OTU24270)  0.39 |

Relative abundance (%, a percentage of the total sequences per sample) of each OTU is under the genus name/group number. OTUs were identified using 97% cutoffs. All Gp4 OTU classify in the same class ‘[Acidobacteria_Gp4](http://rdp.cme.msu.edu/hierarchy/hierarchy_browser.jsp?qvector=204&depth=0&openNode=0&seqid=&currentRoot=2195&searchStr=&endDataValue=&showOpt=)’ and all Gp6 OTU classify in the same class ‘[Acidobacteria_Gp6](http://rdp.cme.msu.edu/hierarchy/hierarchy_browser.jsp?qvector=204&depth=0&openNode=0&seqid=&currentRoot=2195&searchStr=&endDataValue=&showOpt=)’.
