# Supplementary material for: Comparison of the Rhizosphere Bacterial Communities of Zigongdongdou Soybean and a High-Methionine Transgenic Line of This Cultivar
Source: PLoS One. 2014 Jul 31;9(7):e103343. doi: 10.1371/journal.pone.0103343 (PMC4117502; doi:10.1371/journal.pone.0103343)
Supplement: Table S5 — The ten most abundant genera found in the eight samples. Relative abundance (%, a percentage of the total sequences per sample) of each genus is included in parentheses. All Gp6 classify in the same class ‘Acidobacteria_Gp6’, all Gp4 classify in the same class ‘Acidobacteria_Gp4’ and all Gp3 classify in the same class ‘Acidobacteria_Gp3’. (DOC) [file pone.0103343.s005.doc]

Table S5. The ten most abundant genera found in the eight samples

| ZD_1 | ZD_2 | ZD_3 | ZD_4 | ZD91_1 | ZD91_2 | ZD91_3 | ZD91_4 |
| --- | --- | --- | --- | --- | --- | --- | --- |
| Gp4  (23.64) | Gp4  (19.15) | *Sphingomonas*  (11.58) | Gp4  (14.60) | Gp4  (17.68) | Gp6  (14.31) | Gp4  (11.99) | Gp6  (16.12) |
| Gp6  (13.64) | Gp6  (11.99) | Gp4  (11.10) | Gp6  (11.34) | Gp6  (13.17) | Gp4  (11.40) | *Sphingomonas*  (11.51) | Gp4  (11.41) |
| *Sphingomonas*  (5.40) | *Sphingomonas*  (9.44) | Gp6  (7.34) | *Sphingomonas*  (6.16) | *Sphingomonas*  (8.64) | *Sphingomonas*  (5.29) | Gp6  (9.43) | *Sphingomonas*  (4.69) |
| *Gemmatimonas*  (2.62) | *Sphingosinicella*  (3.06) | *Sphingosinicella*  (3.12) | *Terrimonas*  (2.32) | *Sphingosinicella*  (2.60) | *Gemmatimonas*  (2.63) | *Sphingosinicella*  (3.91) | *Lysobacter*  (2.42) |
| *Terrimonas*  (2.31) | *Gemmatimonas*  (2.97) | *Gemmatimonas*  (2.23) | *Lysobacter*  (2.29) | *Gemmatimonas*  (2.57) | *Lysobacter*  (1.73) | *Chryseobacterium* (3.66) | *Gemmatimonas*  (2.26) |
| *Lysobacter*  (1.79) | Gp3  (2.27) | *Lysobacter*  (2.04) | *Gemmatimonas*  (1.88) | *Terrimonas*  (2.13) | *Sphingosinicella*  (1.61) | *Lysobacter*  (3.32) | *Terrimonas*  (1.98) |
| Gp3  (1.55) | Gp7  (1.94) | *Novosphingobium* (1.53) | *Sphingosinicella*  (1.84) | Gp7  (2.04) | Gp3  (1.53) | *Gemmatimonas*  (2.56) | Gp7  (1.70) |
| Gp7  (1.48) | *Flavisolibacter*  (1.89) | *Solirubrobacter*  (1.51) | Subdivision3_genera_incertae_sedis (1.66) | *Lysobacter*  (1.67) | Gp7  (1.20) | *Flavisolibacter*  (2.23) | Gp3  (1.66) |
| *Sphingosinicella*  (1.46) | *Lysobacter*  (1.80) | Gp3  (1.45) | *Flavobacterium*  (1.58) | Gp3  (1.59) | *Flavisolibacter*  (1.15) | *Novosphingobium* (1.54) | Subdivision3_genera_incertae_sedis (1.61) |
| *Ohtaekwangia*  (1.41) | *Terrimonas*  (1.70) | *Flavisolibacter*  (1.31) | Gp3  (1.49) | *Flavisolibacter*  (1.46) | *Steroidobacter*  (1.10) | Gp3  (1.41) | *Sphingosinicella*  (1.58) |

Relative abundance (%, a percentage of the total sequences per sample) of each genus is included in parentheses. All Gp6 classify in the same class ‘[Acidobacteria_Gp6](http://rdp.cme.msu.edu/hierarchy/hierarchy_browser.jsp?qvector=204&depth=0&openNode=0&seqid=&currentRoot=2195&searchStr=&endDataValue=&showOpt=)’, all Gp4 classify in the same class ‘[Acidobacteria_Gp4](http://rdp.cme.msu.edu/hierarchy/hierarchy_browser.jsp?qvector=204&depth=0&openNode=0&seqid=&currentRoot=2195&searchStr=&endDataValue=&showOpt=)’ and all Gp3 classify in the same class ‘[Acidobacteria_Gp3](http://rdp.cme.msu.edu/hierarchy/hierarchy_browser.jsp?qvector=204&depth=0&openNode=0&seqid=&currentRoot=2195&searchStr=&endDataValue=&showOpt=)’.
