# Supplementary material for: Comparison of the Rhizosphere Bacterial Communities of Zigongdongdou Soybean and a High-Methionine Transgenic Line of This Cultivar
Source: PLoS One. 2014 Jul 31;9(7):e103343. doi: 10.1371/journal.pone.0103343 (PMC4117502; doi:10.1371/journal.pone.0103343)
Supplement: Table S6 — Shared OTUs in the samples. (A) Shared OTUs in the ZD libraries. (B) Shared OTUs in the ZD91 libraries. (DOC) [file pone.0103343.s006.doc]

**Table S6 Shared OTUs in the samples**

(A) Shared OTUs in the ZD libraries

|  |  | Shared reads | | | |
| --- | --- | --- | --- | --- | --- |
| phylum | Shared OTUs | ZD_1 | ZD_2 | ZD_3 | ZD_4 |
| Acidobacteria | 244 | 3571 | 2915 | 1555 | 2721 |
| Actinobacteria | 34 | 80 | 94 | 230 | 176 |
| Armatimonadetes | 1 | 8 | 3 | 8 | 9 |
| Bacteroidetes | 73 | 688 | 583 | 433 | 713 |
| Chlorobi | 1 | 1 | 1 | 2 | 2 |
| Chloroflexi | 24 | 196 | 149 | 106 | 242 |
| Cyanobacteria/Chloroplast | 1 | 1 | 2 | 1 | 6 |
| Firmicutes | 15 | 70 | 71 | 84 | 85 |
| Gemmatimonadetes | 20 | 150 | 135 | 68 | 90 |
| Nitrospira | 3 | 19 | 33 | 28 | 14 |
| Planctomycetes | 7 | 24 | 39 | 17 | 24 |
| Proteobacteria | 214 | 1316 | 1803 | 2017 | 1401 |
| unclassified | 47 | 166 | 162 | 122 | 190 |
| Verrucomicrobia | 15 | 54 | 40 | 30 | 61 |
| WS3 | 1 | 1 | 1 | 1 | 1 |
| Total shared sequences | 700 | 6345 | 6031 | 4702 | 5735 |
| Total reads |  | 10762 | 10115 | 9010 | 12250 |
| Shared reads/Total reads (%) |  | 58.96 | 59.62 | 52.19 | 46.82 |

(B) Shared OTUs in the ZD91 libraries

|  |  | Shared reads | | | |
| --- | --- | --- | --- | --- | --- |
| phylum | Shared OTUs | ZD91_1 | ZD91_2 | ZD91_3 | ZD91_4 |
| Acidobacteria | 271 | 3299 | 1991 | 2406 | 2578 |
| Actinobacteria | 41 | 99 | 167 | 175 | 136 |
| Armatimonadetes | 1 | 3 | 4 | 5 | 6 |
| Bacteroidetes | 71 | 753 | 364 | 818 | 578 |
| Chloroflexi | 23 | 204 | 121 | 157 | 147 |
| Firmicutes | 17 | 84 | 111 | 83 | 73 |
| Gemmatimonadetes | 21 | 147 | 110 | 176 | 104 |
| Nitrospira | 4 | 21 | 22 | 26 | 19 |
| Planctomycetes | 7 | 12 | 19 | 14 | 17 |
| Proteobacteria | 192 | 1789 | 1099 | 2292 | 1284 |
| unclassified | 42 | 160 | 158 | 177 | 187 |
| Verrucomicrobia | 10 | 48 | 27 | 22 | 36 |
| Total shared sequences | 700 | 6619 | 4193 | 6351 | 5165 |
| Total reads |  | 12248 | 8556 | 12174 | 12152 |
| Shared reads/Total reads (%) |  | 54.04 | 49.01 | 52.17 | 42.50 |
