# Supplementary material for: Comparison of the Rhizosphere Bacterial Communities of Zigongdongdou Soybean and a High-Methionine Transgenic Line of This Cultivar
Source: PLoS One. 2014 Jul 31;9(7):e103343. doi: 10.1371/journal.pone.0103343 (PMC4117502; doi:10.1371/journal.pone.0103343)
Supplement: Table S7 — AMOVA analysis between ZD and ZD91 in PCoA. AMOVA analysis in PCoA (classfily method: UPGMA). AMOVA analysis was used to find out whether the separation of ZD and ZD91 in the PCoA is statistically significant. (DOC) [file pone.0103343.s007.doc]

**Table S7. AMOVA analysis between ZD and ZD91 in PCoA**

| ZD91-ZD | Among | Within | Total |
| --- | --- | --- | --- |
| SS | 0.080897 | 0.961209 | 1.04211 |
| df | 1.000000 | 6.000000 | 7.00000 |
| MS | 0.080897 | 0.160201 |  |
| Fs: 0.504971 | | | |
| p-value: 0.859 | | | |

AMOVA analysis in PCoA (classfily method: UPGMA). AMOVA analysis was used to find out whether the separation of ZD and ZD91 in the PCoA is statistically significant.
