# Supplementary material for: Comparison of the Rhizosphere Bacterial Communities of Zigongdongdou Soybean and a High-Methionine Transgenic Line of This Cultivar
Source: PLoS One. 2014 Jul 31;9(7):e103343. doi: 10.1371/journal.pone.0103343 (PMC4117502; doi:10.1371/journal.pone.0103343)
Supplement: Table S8 — Primers with tags and adapters used in pyrosequencing. (DOC) [file pone.0103343.s008.doc]

| Sample | Barcode | Forward Primer (515F) | Reverse Primer (926R) |
| --- | --- | --- | --- |
| ZD_1 | ATCGAGCT | CCATCTCATCCCTGCGTGTCTCCGACTCAG-ATCGAGCT-GTGCCAGCMGCCGCGGTAA | CCTATCCCCTGTGTGCCTTGGCAGTCTCAG-CCGTCAATTYYTTTRAGTTT |
| ZD_2 | CAGATAGT | CCATCTCATCCCTGCGTGTCTCCGACTCAG-CAGATAGT-GTGCCAGCMGCCGCGGTAA | CCTATCCCCTGTGTGCCTTGGCAGTCTCAG-CCGTCAATTYYTTTRAGTTT |
| ZD_3 | CGACACAT | CCATCTCATCCCTGCGTGTCTCCGACTCAG-CGACACAT-GTGCCAGCMGCCGCGGTAA | CCTATCCCCTGTGTGCCTTGGCAGTCTCAG-CCGTCAATTYYTTTRAGTTT |
| ZD_4 | CTGATAGT | CCATCTCATCCCTGCGTGTCTCCGACTCAG-CTGATAGT-GTGCCAGCMGCCGCGGTAA | CCTATCCCCTGTGTGCCTTGGCAGTCTCAG-CCGTCAATTYYTTTRAGTTT |
| ZD91_1 | ATGCACGT | CCATCTCATCCCTGCGTGTCTCCGACTCAG-ATGCACGT-GTGCCAGCMGCCGCGGTAA | CCTATCCCCTGTGTGCCTTGGCAGTCTCAG-CCGTCAATTYYTTTRAGTTT |
| ZD91_2 | CATCGATG | CCATCTCATCCCTGCGTGTCTCCGACTCAG-CATCGATG-GTGCCAGCMGCCGCGGTAA | CCTATCCCCTGTGTGCCTTGGCAGTCTCAG-CCGTCAATTYYTTTRAGTTT |
| ZD91_3 | CGTACTCT | CCATCTCATCCCTGCGTGTCTCCGACTCAG-CGTACTCT-GTGCCAGCMGCCGCGGTAA | CCTATCCCCTGTGTGCCTTGGCAGTCTCAG-CCGTCAATTYYTTTRAGTTT |
| ZD91_4 | TACGACTG | CCATCTCATCCCTGCGTGTCTCCGACTCAG-TACGACTG-GTGCCAGCMGCCGCGGTAA | CCTATCCCCTGTGTGCCTTGGCAGTCTCAG-CCGTCAATTYYTTTRAGTTT |

**Table S8. Primers with tags and adapters used in pyrosequencing**
